# Trust in the early chain of healthcare: lifeworld hermeneutics from the patient’s perspective

**DOI:** 10.1080/17482631.2017.1356674

**Published:** 2017-08-10

**Authors:** Gabriella Norberg Boysen, Maria Nyström, Lennart Christensson, Johan Herlitz, Birgitta Wireklint Sundström

**Affiliations:** ^a^ Faculty of Caring Science, Work Life and Social Welfare, University of Borås, PreHospen – Centre for Prehospital Research, Borås, Sweden; ^b^ Faculty of Caring Science, Work Life and Social Welfare, University of Borås, Borås, Sweden; ^c^ Department of Nursing, School of Health and Welfare, Jönköping University, Jönköping, Sweden

**Keywords:** Ambulance, healthcare centre, healthcare level, caring relationship, caring science, trust, lifeworld hermeneutics

## Abstract

**Purpose**: Patients must be able to feel as much trust for caregivers and the healthcare system at the healthcare centre as at the emergency department. The aim of this study is to explain and understand the phenomenon of trust in the early chain of healthcare, when a patient has called an ambulance for a non-urgent condition and been referred to the healthcare centre.

**Method**: A lifeworld hermeneutic approach from the perspective of caring science was used. Ten patients participated: seven female and three male. The setting is the early chain of healthcare in south-western Sweden.

**Results**: The findings show that the phenomenon of trust does not automatically involve medical care. However, attention to the patient’s lifeworld in a professional caring relationship enables the patient to trust the caregiver and the healthcare environment. It is clear that the “voice of the lifeworld” enables the patient to feel trust.

**Conclusion**: Trust in the early chain of healthcare entails caregivers’ ability to pay attention to both medical and existential issues in compliance with the patient’s information and questions. Thus, the patient must be invited to participate in assessments and decisions concerning his or her own healthcare, in a credible manner and using everyday language.

## Introduction

This study is one part of a major research project intended to deepen our knowledge about a healthcare model named “Right Level of Care”. The overall aim of this research project is to explore whether ambulance nurses can assess and determine whether patients with non-urgent conditions can be referred directly to the healthcare centre (HCC) with maintained medical safety, and how patients experience trust in the early chain of healthcare in these particular situations.

The present study is one of a series of studies that started with a retrospective study of patient records to explore the population of patients with non-urgent conditions from the perspective of the emergency medical services (EMSs) (Norberg, Wireklint Sundström, Christensson, Nyström, & Herlitz, 2015). This was followed by a study developing an instrument to measure patient trust: the Patient Trust Questionnaire (Norberg Boysen et al., 2016). This present study investigates the patient’s lived experience of trust in the early chain of healthcare including three frontline service providers: the dispatch centre, the ambulance services (EMSs) and the HCC (primary healthcare).

### The early chain of healthcare

The early chain of healthcare is part of the public health service. It includes the dispatch centre and the handover to the receiving healthcare facilities, including ambulances, other emergency vehicles and helicopters (Suserud, Bruce, & Dahlberg, 2003a, 2003b). The receiving unit may be either the emergency department (ED) or the HCC.

Research points out the advantages of helping patients to an optimal level of healthcare directly (Hjälte, Suserud, Herlitz, & Carlberg, 2007; Johansson, 2006). Alternative destinations to traditional ED care have been shown to have many potential benefits for patients and the general healthcare system, and to reduce the burden on the ED as well (Nutting, Goodwin, Flocke, Zyzanski, & Stange, 2003; Olshaker, 2009; Snooks et al., 1998; Weinick, Burns, & Mehrotra, 2010). It seems reasonable to assume that alternative destinations in general shorten both transport and waiting times (Nutting et al., 2003; Snooks et al., 1998).

Referring all patients who call for an ambulance to the ED is an incorrect use of medical resources. In many cases, the patient’s needs are met through increased cooperation between the ambulance services and the HCC (Porter et al., 2007; Snooks et al., 2004; Vicente, Svensson, Wireklint Sundström, Sjöstrand, & Carsten, 2014). An alternative to traditional healthcare at the ED may thus include referring patients with minor illness or injury to the HCC (Coughlan & Corry, 2007; Muntlin Athlin, Von Thiele Schwarz, & Farrohknia, 2013; Olshaker, 2009). There are national directives in Sweden that require collaboration between various healthcare organizations (SFS 1982:763). However, there are no guidelines on how this should be enforced in practice. Furthermore, there are trends showing that patients have greater confidence in the ED than in the HCC (SKL, 2011), which may affect the choice of healthcare provider.

Although these options have advantages in terms of efficiency, few studies have been conducted in which patients were asked about how they reacted to an alternative level of care to the ED, when seeking help for non-urgent conditions (Jones, Wasserman, Li, & Shah, 2015; Munjal et al., 2016). Thus, the proportion of patients supporting transportation to an alternative destination has been reported to range between 58% (Munjal et al., 2016) and 69% (Jones et al., 2015). To our knowledge, no one has ever asked about the patient’s lived experiences of the early chain of healthcare in this particular situation.

### Trust in the early chain of healthcare from the patient’s perspective

There is disagreement about how to define trust, even within single disciplines (Hupcey, Penrod, Morse, & Mitcham, 2001; Pearson & Raeke, 2000), but trust is well-defined as an important concept in all caring disciplines (Hupcey et al., 2001). Hupcey et al. (2001) consider that trust exists when someone decides to place her or himself in a dependent or vulnerable position. It is not based on any risk assessment from the patient’s perspective. Theoretically, trust generates a context in which patients give valid and reliable information (Hagerty & Partusky, 2003). Norberg Boysen et al. (2016) found that credibility and availability are underlying dimensions of trust, especially in the early chain of healthcare. Few studies have highlighted the patient’s perspective on trust in this context (Norberg Boysen et al., 2016). Therefore, it is of interest to understand the meaning of trust with openness from the patient’s perspective.

The aim of this study is to explain and understand the phenomenon of trust in the early chain of healthcare, when a patient has called an ambulance for a non-urgent condition and been referred to the HCC. In this study, the optimal level of healthcare means care at the level that is most appropriate and still as limited as possible with maintained patient safety.

## Methods

A lifeworld hermeneutic approach was chosen (Dahlberg, Dahlberg, & Nyström, 2008; Nyström, 2016). Since hermeneutics are often associated with interpretations of different texts (Palmer, 1972), it is worth noting that the approach chosen here is based on the hermeneutic thinking that Gadamer suggested after Husserl’s introduction of the “lifeworld” concept. Warnke (1995) talks about this as a phenomenological development of hermeneutics. An epistemological foundation in the lifeworld thus means that research is focused on the lived experiences of a phenomenon, not on the participants in the study. An interpretative analysis inspired by Gadamer (1997) and Ricoeur (1976) has been used to suggest how to explain and understand lived experiences of the phenomenon of trust when a patient calls an ambulance for a non-urgent condition and is referred to the HCC. Epistemologically, the study is based on the lifeworld perspective introduced by Husserl and developed further by Gadamer’s hermeneutics, emphasizing an open approach when trying to understand something new. This has meant that the authors, with their professional experiences from the care context under study, have made every effort to understand what Gadamer calls “otherness” in data (Gadamer, 1997), i.e., something different from their own preunderstanding. The theoretical support used here for the main interpretation was not chosen in advance, but only selected after the open interpretations had been formulated and considered valid. To develop the interpretations further, the analysis was also stimulated by Ricoeur’s proposal that new understanding often builds on a certain amount of explanation (Ricoeur, 1976).

### Context

The context and study setting is the early chain of healthcare in the south-west of Sweden, consisting of the dispatch centre, the ambulance services and the HCC.

#### The dispatch centre

By assignment of the Swedish Government, the dispatch centre is responsible for the emergency number 112. The staff at the dispatch centre receive, coordinate and relay the alarm to ambulance, police and fire rescue. As support for the operators, there are dispatch nurses for more complex medical cases (Regeringskansliet, 2015).

#### The ambulance services

According to a regulation from the Swedish National Board of Health and Welfare, ambulance crews always include at least one registered nurse, often an ambulance nurse, specialized in prehospital emergency nursing, and one medical technician/paramedic or an assistant nurse (Suserud, 2005). The registered nurse/ambulance nurse has the overall responsibility for prehospital care, medical assessment, medical treatment and other care aspects such as caring relationships with patients and relatives (Holmberg & Fagerberg, 2010).

#### The HCC

The HCC is part of the public healthcare service and is responsible for patients’ basic medical treatment, prevention and rehabilitation not requiring the hospitals’ medical and technical resources or expertise. The staffing varies, but commonly includes assistant nurses, registered nurses, primary healthcare nurses and general physicians (Socialstyrelsen, 2016).

### Participants and data collection

The participants in this study were patients who had called the dispatch centre, been assessed by ambulance nurses as having non-urgent conditions and then been referred to the HCC. They all participated in an intervention study evaluating the healthcare model, named “Right Level of Care”.

All patients in the intervention study were contacted by the first author, and 10 of them were selected for an interview. The selection of participants in the present study was purposeful and aimed at optimizing the variation in the phenomenon of trust in the early chain of healthcare, regarding age, gender and ethnicity of the population in the area. All 10 patients (seven female and three male, age range 20–87 years old) approached agreed to participate. Nine were of Swedish descent and one had foreign origins. All were Swedish speaking. Five lived in an urban and five in a rural area. The participants chose the time and place for the interviews. Seven interviews were conducted in the patients’ homes, one at a neutral site and two at the university.

The interviews followed the principles of an open lifeworld approach (Dahlberg et al., 2008). The participants were invited to describe their lived experiences of the current situation, i.e., including all three frontline service providers. The initial question was the same in all interviews: “How was it when you called an ambulance and were referred to the HCC?” Depending on individual responses, the probing questions varied, but they all aimed at stimulating reflection on the research phenomenon, for example, “What did you think about that?” or “How did you feel then?” The interviews were recorded and then transcribed verbatim.

### Data analysis

The initial part of the analysis involved reading the transcribed interviews several times until the whole data set became familiar. In the next step, five themes that suggest how to explain and understand different aspects of the research phenomenon were identified and further described and interpreted, using a dialectical structure with opportunities and obstructions for trust to occur. In each theme, prerequisites for trust are placed against obstacles for trust as a thesis against an antithesis. The subsequent synthesis consists of an interpretation that suggests how the phenomenon of “trust” in the early chain of healthcare, when a patient has called an ambulance for a non-urgent condition and has been referred to the HCC, can be understood. The emerging interpretations were evaluated using the following validity criteria (Trankell, 1973):

- An actual piece of data (a meaning unit) must constitute the only source of an interpretation.
- No other interpretations should be more meaningfully able to explain the same data.
- There must be no contradiction in the data upon which interpretations considered valid are based.

The final step was a comparison of the themes interpreted, using comparative analysis. This was followed by a main interpretation suggesting how the phenomenon might be understood. Mishler’s (1984) theory of the medical voice versus the lifeworld voice turned out to be able to spread further light on the five themes that were interpreted according to the principle of openness. According to Mishler (1984), caregivers and patients usually have different perceptions of what is important in a healthcare relationship. Caregivers often speak only with the “voice of medicine”, but are deaf to the patient’s existential questions. Thus, patients often try to adapt to their caregivers’ “voice”, with the result that their lifeworld remains ignored. Consequently, caregivers only acquire a fragmented picture of the patient’s problems (Mishler, 1984).

The validity of the main interpretation was assessed in relation to the principle of moving from the initial whole (data set) to parts (interpretations and comparison of the themes) to the new whole (main interpretation) and *vice versa*, striving for consistency in the structure of interpretations (Ödman, 1994). To ascertain that the first author did not in any way influence the interpretations, the third co-author, without experience in prehospital emergency care, carefully compared all of the interpretations with the data set.

### Ethical considerations

Permission and approval to conduct the study were obtained from the Research Ethics Committee of the Medical Faculty at the University of Gothenburg (registration number 329-12). All participants received both written and oral information and gave their written consent. They were also informed that participation was voluntary and that they could interrupt the interview whenever they wanted. They were also guaranteed complete confidentiality (World Medical Association Declaration of Helsinki, 2008). There is no reason to believe that these patients were particularly vulnerable or sensitive in relation to the current interview questions. However, the first author stayed on the scene for a while after the interviews had been completed to ensure that the patients felt well.

## Results

The results consist of Parts I and II. In Part I, the five themes are interpreted based on the dialectical structure. For clarity, each theme is structured as opportunities and obstructions, followed by a synthesizing interpretation.

In Part II, a comparative analysis where all five themes are interwoven is followed by a main interpretation that suggests how trust in the early chain of healthcare can be explained and understood when a patient has called an ambulance for a non-urgent condition and been referred to the HCC (Table I).

**Table I.** Themes and main interpretation.

Themes	Main interpretation
Perceiving professional experience and healthcare competence	Trust in the early chain of healthcare does not automatically involve medical care. However, attention to the patient’s lifeworld in a professional caring relationship enables the patient to trust the caregiver and the healthcare environment
Acquiring information and being allowed to communicate one’s problems	
Meeting a welcoming and inviting healthcare atmosphere and environment	
Observing and recognizing fair healthcare	
Understanding one’s own responsibility	

### Part I

#### Perceiving professional experience and healthcare competence

Patients’ perception of caregivers from the three frontline service providers as having adequate competence, knowledge and previous experience of similar care situations, allows patients to feel secure in handing themselves over into the professional hands of caregivers. This is more about a feeling that the caregivers know what they are doing, than about what caregivers actually say and do. Knowledge also involves the art of coping with people. Caregivers are assigned the role of being special experts, and the patient must be able to rely on both caregivers and the healthcare facility:

Maybe I should’ve asked what the ambulance nurse thought. I’d most likely have trusted their expertise. … I’m sure they know more about it [level of care] than I do.

When competence and experience seem to characterize professional healthcare as a whole, the patient trusts healthcare providers to provide adequate help that makes him feel secure:

Feeling safe means being able to trust that they will help me.

Sometimes it is difficult for patients to dare to rely on caregivers’ competence, knowledge and skills. This is particularly evident when a patient questions a caregiver’s assessment of the optimal care level in her individual case. In the example below, a patient who was sent on a secondary transport to the ED that she had not expected did not understand why this transport from the HCC was necessary:

That was not so good because of course I thought they could assess it down here at the healthcare centre.

When caregivers do not do what they have promised, trust is lost and replaced with distrust regarding the caregivers’ reliability:

The doctor at the healthcare centre was going to get in touch about the samples and all that, but they haven’t done so … of course one wants to be able to trust what they say.

Thus, an important aspect of experiencing trust is when caregivers invite the patient to a dialogue that requires communication in a non-dominant way. It seems, moreover, to be essential for caregivers to pay attention to patients’ expectations. In the following example, the patient does not receive the expected attention, and this immediately reduces his trust in the physician’s competence:

Shit, when the doctor was in here it all happened so quickly. He came in and checked me over a little … then he said that I could go home. He didn’t even ask if I needed sick leave. … It was as if I didn’t exist—I don’t think he was any good.

#### Acquiring information and being allowed to communicate one’s problems

During the whole chain of healthcare, it is important to be given early and appropriate information that is easy to understand and individually tailored. This implies that patients must be able to understand what is said, which may involve volume and cognitive or linguistic customization. Effective information must be presented simply and in an understandable way to optimize the patient’s chances of feeling convinced that important information has not been left out and that he has understood the healthcare process being presented:

Here they spoke slowly and clearly. … They made sure that I understood by asking me and looking at me.

Opportunities for providing information may also include occasions for the patients to share their problems with caregivers. For this to happen, the patient must feel trust towards the caregivers, otherwise they will not want to share his problems:

It’s like offloading your worries somehow. The ambulance takes care of everything … well, not everything perhaps, but anyway it feels much better.

Being invited into the dialogue with the caregivers and being involved in decisions are good experiences for patients. One example is to give patients time to describe clearly their perceived healthcare problems in their own words. This approach helps the patient to feel involved:

I felt much better describing my troubles myself rather than just sitting and looking on, in a corner, while the ambulance nurse described them …

Communication problems may create barriers in caring relationships. Some examples are language differences or a language use that distances patients and caregivers from each other. Lack of, or inadequate information may also create fear arising from the patient’s loss of control. Such feelings often lead to loss of trust in caregivers:

Some of the doctors one has met are so bloody self-important and one can’t understand a word they’re saying. They talk as if they come from another planet, you see. Not all of them know Swedish either … If one needs help, one must be able to understand what they’re trying to say.

Information that is understandable, comprehensive, accessible and clear is essential for promoting trust in the early chain of care. Sharing problems with caregivers is an important aspect of truthful communication between the caregiver and the patient, and this requires an open attitude and frank behaviour from the caregiver:

The ambulance staff were damn good at talking so one understood, and they joked a little too … and that way it’s not all so bloody stiff and starchy either.

#### Meeting a welcoming and inviting healthcare atmosphere and environment

When the caregivers and the healthcare environment are experienced as familiar, at least to some extent, patients feel strengthened. Feelings of support and well-being emerge from the experience of being seen and listened to, and the atmosphere seems warm and welcoming. This personal relationship allows the patient to experience caregivers and the healthcare environment as pleasant, which in turn positively affects the perception of the whole care situation. Being recognized by the caregiver in “small” healthcare environments underlies the experience of being invited to participate in a caring relationship:

I prefer the healthcare centre. … I think you get treated better at a healthcare centre, because it’s smaller and it feels as though people are a bit kinder …

An HCC is generally easier to gain an impression of than the ED, since it is easier to see what is happening in the whole area. The limited dimensions of the environment reduce the risks of being neglected as an anonymous number or of being forgotten in a crowd of patients. The benefits of a small, intimate healthcare environment become especially clear when it is compared with large healthcare environments such as EDs, where the patient often feels abandoned, invisible and forgotten. Difficulties in grasping the external situation seem to constitute an obstacle to a trustful relation:

So you’re more visible in a small room than in a large one. … It feels a little cosier and as though they listen to you more [HCC]. … At the emergency department there are just so many people on the move all the time and it’s all too easy to be just left sitting there and one gets the feeling that they are not listening to one.

Thus, an important aspect of trust in the early chain of care has to do with the recognition of a familiar atmosphere. The experiencing of such an atmosphere need not mean that the patient has actually attended the HCC in question before, but that a welcoming and inviting care environment can create feelings of familiarity even in places where the patient has never been before:

I’d never been in that situation before. … It was strange to drop one’s guard and just let somebody look after one. … The nurse explained very clearly exactly what was going on, … so that one grasped what it was anyway.

#### Observing and recognizing fair healthcare

If the healthcare organization is experienced as fair, making no distinction between patients, then patients dare to trust that all important requirements will be fulfilled in an appropriate and careful manner. The patient then feels valued and she feels that adequate care and attention are guaranteed when needed:

I feel that they do listen to everybody and that they are somehow always gentle and careful. I don’t feel that they make any difference between if it’s an old lady or if it’s the prime minister.

An important aspect of fair and professional attention is to be allowed to take one’s own time to gain sufficient attention from the professional caregiver. Enough time allows the patient to feel that he has some control over the situation:

An old person isn’t that quick, whether in thought or in body, so it’s good if you’re given a chance to keep up … and I was given that chance.

The importance of fair healthcare seems to be even more evident when the opposite is true. Then, patients feel neither noticed nor supported. On such occasions the patient feels that he is not allowed just to be in need of care, and may experience being unfairly treated. Emotions such as jealousy and frustration may be expressed in terms of anger:

Four people were allowed to go in before me even though they arrived in the healthcare centre’s waiting-room after me. That made me angry, like, and I thought that nobody should be treated in such a bloody rotten way ….

An important aspect for creating trust in the early chain of care is clearly the principle of fairness: a healthcare characterized by equal right and access to healthcare, where the medical assessment takes precedence. This means that time and resources must be sufficient for everybody, and that all requirements must simultaneously be assessed and adapted according to individual needs. All patients have the right to respectful treatment, medically and socially, where their needs are met. This in turn gives a sense of security grounded on the belief that adequate and fair healthcare is available to all who need it:

People shouldn’t complain so much about healthcare, whatever anyone says it’s pretty good in Sweden. People needing care do get it.

#### Understanding one’s own responsibility

Patients feel satisfaction after making correct decisions as to where and how to seek healthcare. Awareness of the severity of one’s own illness or injury enables one to feel more comfortable with the experienced condition. The patient also begins to understand others’ healthcare needs and a sense of loyalty towards fellow sufferers may awaken in her:

There were many people there in much worse condition than me.

Patients’ insight concerning their own responsibility will, it is hoped, lead to recognition that healthcare resources must be utilized properly. An understanding of how to seek healthcare and how resources should best be utilized may lead the patient to feel a sense of responsibility for her own contact with healthcare:

I spoke to the healthcare advisory service and they told me their analysis of my symptoms and that certainly helped me to come to a decision.

On the other hand, patients may feel that their healthcare contact, such as the healthcare advisory service, is taking their illness or injury too seriously. In such cases, the patient dare not do anything other than follow their recommendation. In retrospect, however, realization may dawn that the resources offered were not really needed and feelings of guilt and shame may follow:

I did think that it was rather drastic, I didn’t think that I was so ill as to need an ambulance. … Perhaps, I could’ve got a lift with somebody.

Thus, patients gain new insights into their own responsibility, demonstrating another important aspect of trust in the early chain of healthcare. Patients seem to search for meaning, while trying to understand how the healthcare system works. There is a genuine desire to try to do right and adjust to the rules that apply in healthcare, even though these rules are sometimes difficult to understand for the uninitiated. Understanding how healthcare works and the feeling of being able to take responsibility for her own contacts with healthcare seem to engender loyalty to other patients:

It was “overkill” to some degree to call the ambulance initially, but I didn’t know that. I think that the healthcare advisory service should have said: “go to your HealthCare Centre”.

### Part II

Primary healthcare may be experienced as familiar, in contrast to care at the ED, whether it comes as a positive surprise or a frustrating disappointment. Interpersonal relations and the healthcare environment as a whole play an essential role for the emotions dominating the overall experience. Being invited to participate in decisions concerning his or her own healthcare enables the patient to feel involved and supported. The need for care is thus allowed and accepted.

#### Comparative analysis

When the five synthesizing interpretations above are compared and interwoven, a pattern emerges showing that trust is based on several interpersonal aspects. One important part of this is how caregivers inform the patient about decisions, and how the patient assesses the caregivers’ competence to make such decisions. A good starting point for further assessments in the early chain of healthcare is to give the patient sufficient information and to allow him or her adequate involvement early on in the process. It is worth noting, however, that adequate involvement can mean anything from active participation to putting oneself in safe professional hands.

At the handover to the HCC, the patient’s trust needs to be secured once again. If the receiving facility is well known and is perceived as familiar, the patient’s trust in the early chain of healthcare is relatively quickly restored. When the current HCC is previously unknown, the quality of the first meeting becomes increasingly important. Trust now seems to be based on this particular situation and the patient’s possibilities of gaining an overview of the whole surroundings. Whether the patient is aware of the environment or not, a welcoming caring relationship includes early information and time for him or her to relate what prompted the decision to call the dispatch centre. Feeling safe increases the patient’s chances of understanding and experiencing control of what is happening. Experiencing thorough and fair treatment also appears to stimulate the understanding that other patients sometimes need to be prioritized before oneself.

#### Main interpretation

The phenomenon of trust in the early chain of healthcare does not automatically involve medical care. However, paying attention to the patient’s lifeworld in professional caring relationships enables the patient to trust caregivers and the healthcare environment (Table I). Secure medical healthcare and treatment seems, in fact, to be perceived as something to be taken for granted, provided that healthcare problems are listened to and understood by caregivers. Being involved in the decisions about coming to the HCC in a way that suits the patient’s own individual needs strengthens this positive spiral further.

The core in this professional caring relationship is described as being shared communication in the form of information, dialogue and discussions. Being invited to tell his or her stories increases the possibility for the patient’s trust and security to develop into insight about healthcare at the right level. This in turn increases the chances that the patient will dare to seek care at a more limited level in future if similar symptoms appear. One possible reason for this is that building the patient’s trust in the early chain of healthcare requires care situations getting off to a positive start. When this happens, the concept of care can be expanded to include medical care and treatment as well.

The meaning of this caring relationship and communication can be further illuminated by Mishler’s (1984) theory of “voices”. Mishler (1984) means that the patient’s medical questions should be answered using the “voice of medicine”, while existential issues should be answered using the “voice of the lifeworld”, i.e., with open follow-up questions that comply with the patient’s own ways of describing problems and needs. Mishler’s (1984) theory highlights the importance of avoiding the objectification of the patient in all kinds of situation. Using the right voice in each situation enables the patient to feel trust and involvement in his or her own healthcare.

The voice of the lifeworld in a professional caring relationship thus seems to enable the patient to trust the caregiver and the healthcare environment. To put it another way, when caregivers in the early chain of healthcare invite the patient to participate in assessments and decisions concerning his or her own healthcare, in a credible manner and using everyday language, this produces trust in the patient.

## Discussion

### Methodological reflections

This study has followed the principle of the hermeneutic circle, which represents a movement between inner reflection and outer tentative dialogue with the data, in the development of an understanding horizon. This means an oscillation between the whole (data set) and the parts (interpretations and comparison of the themes) and back to a new whole (main interpretation) to gain a deeper understanding, in this case of trust in the early chain of healthcare. Gadamer’s (1997) horizon fusion explains where a new form of knowledge arises in relations, in meetings between people. This does not imply the changing or taking over of opinions but rather, as shown in this study, the importance of listening to and considering all opinions and trying to understand. For this “fusion of horizons” to occur, caregivers must be able to use the voice of the lifeworld. Lifeworld hermeneutics focus on the meaning of lived experiences and the results should be understood as a proposal for how the phenomenon of trust itself, and the conditions for trust to occur, can be created in a particular context.

### Reflections on the findings

The results suggest that trust in the early chain of healthcare, after calling an ambulance for non-urgent conditions and being referred to an HCC, does not automatically include medical care. Instead, the professional caring relationship appears to be the most significant issue, to the extent that medical treatment was not mentioned at all when the participants were asked about their experiences. Similar situations have been discussed by Abrahamsson, Berg, Jutengren, and Johnsson (2015) and they state that even if the medical treatment is successful, it cannot compensate for the lack of a caring approach, because perceptions of a care situation are not limited to the treatment situation. The patient probably takes into account several impressions during all stages of the healthcare process, including accessibility, convenience and the feeling of being well received by the caregivers. Corresponding results have also been demonstrated regarding trauma patients’ perceptions of nursing care (Berg, Spaeth, Sook, Burdsal, & Lippoldt, 2012). On the other hand, unsatisfactory caring relationships may result in a loss of trust, even if the medical treatment itself is been successful, which is in line with Bultzingslöwen, Eliasson, Sarvimäki, Mattsson, and Hjortdahl (2006).

The present results imply that positive and respectful caring relationships with caregivers enable patients to feel involved in any decisions made. Professional caring relationships must be reflective and focus on the patient and his or her needs. Thus, the patient must also be given enough time to explain their individual health conditions. The aim of such an approach is for caregivers to understand what is required to support the patient’s health processes (Todres, Galvin, & Dahlberg, 2014). Through conversations and dialogues, caregivers can support the patient’s health processes by helping them to come to an understanding of healthcare culture. This may mean merging the voice of medicine and the voice of the lifeworld. With the help of the voice of the lifeworld, patients can experience inclusion and affirmation. The understanding and feeling of being in control can develop, which may in turn stimulate awareness of and consideration towards fellow patients. Todres et al. (2014) argue that from a caring sciences perspective, this means affirming the patient’s lifeworld and participation. Caring relationships may help patients to understand themselves and the situation, and thereby strengthen their capability and power to seek care at the right level, as shown in this study. Further research has shown that continuity among caregivers also increases opportunities for winning the patient’s trust, by increasing understanding of individual needs (Abrahamsson et al., 2015; Bultzingslöwen et al., 2006; Redsell, Stokes, Jackson, & Baker, 2007).

According to the present results, it seems fair to suggest that it is possible to feel trust even if the caring relationship is short, as in an ambulance transport to the HCC. These results have been confirmed by Holmberg, Forslund, Wahlberg, and Fagerberg (2014). They argue that trust in a prehospital setting can be created in a caring relationship even if it is brief. The results have also been confirmed by a previous study (Sikma, 2006) dealing with the topic of how the external environment affects the patient’s experiences of trust. In this study, interpersonal trust and trust in the healthcare environment coincide with each other. However, Pearson and Raeke (2000) do not agree with the present results but argue that interpersonal trust needs time, and claim further that it is important to distinguish between social trust and interpersonal trust in a healthcare context. They state that social trust is trust in a particular institution, i.e., trust in the specific healthcare environment, while interpersonal trust is trust in the caregiver.

The present study suggests that a caring relationship with more nuanced communication, i.e., more focus on the voice of the lifeworld (Mishler, 1984), may help the patient to experience trust more easily. As things are, especially in the early chain of healthcare settings, the risk is great of patients trying to adapt to the voice of medicine and current healthcare culture, thus damaging their own chances of being able to signal their own needs and conditions, and their desire to speak with the voice of the lifeworld. The patient attempts to adapt to the culture of healthcare and say what he or she believes the caregivers want to hear, depriving caregivers of the opportunity to interpret the patient’s lifeworld. Thus, without interpretation, no professional caring relationships can emerge. However, earlier research points out that a certain risk for paternalism does exist in healthcare, which can silence the lifeworld voice (Lynöe, Juth, & Helgesson, 2010). Therefore, it is important for the patient to be treated personally and to receive clear explanations that can be understood (Theis, Stanford, Goodman, Duke, & Shenkman, 2016). Even Eldh, Ekman, and Ehnfors (2006) suggest that caregivers can improve the quality of healthcare significantly through empathy, good communication and attention to individual needs. From this perspective, we can gain insights into how to relate to ourselves and others.

## Conclusion and clinical implications

Trust in the early chain of healthcare entails caregivers’ ability to pay attention to both medical and existential issues in compliance with the patient’s information and questions. This involves inviting the patient to participate in assessments and decisions concerning his or her own healthcare, in a credible manner and using everyday language, since this arouses trust in the patient.

Creating trust in the early chain of healthcare is based on patient–caregiver relations and an environment that involves all caregivers communicating with the patient in ways that affirm diversity and individuality. It also involves creating conditions for trust even when care is not provided at the institution where a patient first expected to receive it. Two noteworthy results in these circumstances are, first, that the patient generally experiences the care given as fair and just anyway; and secondly, that taking trouble to build up a trustful relationship is no more time consuming than not doing so. This also involves the understanding that patients who are more unwell must take precedence. Personal insights into the advantages of attending primary healthcare when not requiring immediate emergency care are, in the longer term, valuable for both the patient and society.

The results of this study could lead to new possibilities for caregivers to establish professional caring relationships with patients who have called an ambulance for non-urgent conditions, and further enable caregivers to help the patient to choose the optimal level of care in each particular case. Caregivers must be aware of how crucial it is to relate to and communicate with the patient. Since patients and caregivers look at healthcare issues from different perspectives, caring competence, in addition to medical competence, is involved. Therefore, based on the findings, we suggest the following clinical implications for creating trustful relations with the patient:

- Offer a welcoming atmosphere to awaken the patient’s insight about healthcare at the right level.
- Strive to give healthcare a positive start by using everyday language.
- Invite the patient to participate in assessments and decisions.
- Offer the patient individualized space to communicate her or his healthcare needs.
- Involve the existential aspects of healthcare as well as medical care and treatment.

If these measures are fully implemented, the benefits for the patient, the early chain of healthcare and the overall healthcare system should be that:

- No additional time will be needed for the patient–caregiver relationship.
- The patient can rely on getting access to more urgent measures if necessary.
- The patient will dare to seek care at a more limited care level in future if similar symptoms appear.

### Continuing research

Suggestions on continuing research involve discovering appropriate methods to implement the voice of the lifeworld, i.e., to pay attention to the patient as a feeling, thinking and acting human being, in the early chain of healthcare. The findings are probably transferable to other healthcare contexts, but further research is needed.
